# "What Can I Do to Help My Country?"

**Published:** 1914-09-19

**Authors:** 


### The Hospital

The Workers' Newspaper of Administrative Medicine and Institutional Life, Administration, National Insurance and Health.

No. 1474, Vol. LVI. SATURDAY, SEPTEMBER 19, 1914. PRICE ONE PENNY.

### "WHAT CAN I DO TO HELP MY COUNTRY?"

### Defend the Families of Our Sailors and Soldiers.

This question is being answered by some of the younger and nobler men amongst us who are flocking to the Colours in response to Lord Kitchener's appeal. England needs a million such men, of whom 500,000 have already joined. The remaining 500,000 and many more could be recruited within a fortnight if each British citizen who stays at home would make a vow, and keep it, that no dependant of any man who is fighting our battles shall go hungry while he and his have bread to eat. Every man who joins the Colours makes a grave personal sacrifice, and risks his life for the good of the nation and the protection of each one who remains behind. The personal risk does not cause a single live man eligible to become a soldier to hesitate, but when he is the bread-winner upon whose earnings a wife and children, a mother and younger brothers or sisters depend, he cannot properly go to the war and leave his dependants without material support. There are some millions of men of mature age in comfortable circumstances, to whatever class of society they may belong, who are ineligible to join the Colours. Every one of these men is bound as a matter of sentiment, of justice, of self-protection, and of business gladly to agree to provide the money necessary to free every recruit from all anxiety as to his dependants during his absence, or should he lose his life or become permanently disabled through the war. This sound principle must be recognised, and by adopting it every male resident in the United Kingdom worthy of the name of man will do his part to help his country. In the circumstances of the moment the British are in the proud position of fighting in a just cause for the vindication of the rights of small States, the enforcement of the public law of Europe, and the assurance of its lasting peace. Great as were the opportunities of our forebears and sires, nobly as they fulfilled their duty in the years that are past, their descendants to-day not only enjoy a noble heritage but have an infinitely greater opportunity to cement indissolubly the unity of the British Empire and the peace of the world than has ever been presented since the world began.

It follows that we ought to be justified in taking for granted that both our sailors and soldiers have already secured as a right through the Government an allowance for their dependants sufficient for the maintenance of a standard of comfort and the upkeep of the home during each warrior's absence. Unfortunately, as the *Daily Chronicle*, to its infinite credit, pointed out in a leader on the 14th instant, unthinkable though it be, our sailors' and soldiers' dependants still have inadequate Government allowances eked out by charitable doles. Not only has this been so, but cases have arisen where the families of men who have joined the Colours have received Poor-Law relief. Indeed, a circular, dated 3rd instant, issued by the Local Government Board intimates that the Army Council are prepared to issue separation allowances and allotments monthly in advance to a charitable association or the local representative (distress) committees, to be handed by these bodies weekly to the dependants of our sailors and soldiers at the front. How is it that the Army Council has so failed to catch the present spirit of the people of these islands and of the British Empire that it should thus fail to uphold the independence of the wives and children of the warriors who are fighting our battles at the front? The Acting Secretary of the Charity Organisation Society, in a letter we publish in another column, confirms the wide-spread adoption of this plan to treat the representatives of some of the noblest types of the British nation as objects of charity and distressed persons. Anything more monstrous or improper it is impossible to conceive. Mr. Bonar Law, as we showed last week, pledged himself, on behalf of the politicians not only of his own party but of all parties, that it should be recognised that our sailors and soldiers are children of the State, and that they have the first claim upon the resources of our nation. Fancy what Charles Dickens would say if he were alive when he learnt that, so far from being treated as children of the State, the dependants of our sailors and soldiers are being handed over to the administrators of the Poor Law, to the managers of charities, and to Distress Committees. This grave disgrace to every man amongst us must be wiped out and put an end to at once. All thinking people who have any brains to realise the gravity of the evils to which we here call attention will join with us in insisting upon the literal fulfilment of the implied promise made by the political leaders who spoke at the Guildhall on the 4th instant.

The amount of money involved in relation to the total expenditure which the war will require, though serious, is one which can well be provided, as Mr. Robert Blatchford ably explains in the *Weekly Dispatch* of the 13th instant. Assume that every one of us who pays income tax has to provide an extra 1s. in the £ to make the necessary provision for the dependants of our sailors and soldiers under all the circumstances we have indicated. The stay-at-homes each time they pay their income tax in future will be able to feel that they have directly contributed to the splendid results which this war, when brought to a victorious conclusion, as it must be brought, will confer upon humanity by the perpetuation of peace amongst nations and of freedom and protection to all the peoples, however relatively few in numbers a nation may be. All the existing evils and mistakes can be remedied in the simplest possible manner. Every payment to every dependant must be made through the Post Office. The Lord Mayors, the Mayors, the chairmen of County and District Councils, and the head of the local authorities throughout the kingdom will invite (*a*) householders of good faith to enter their names upon a Register of friends of our sailors and soldiers, and (*b*) each dependant in receipt of the Government allowance to send her name and address to him in writing, if she would like to have a friend to refer to. With the Government allowance and the co-operation of such a friend through the Lord Mayor or other authoritative person the dependants of every sailor and soldier will be enabled to keep up the home, and to maintain themselves in comfort during the absence of their dear one on service with the Colours.

In this way every taint of charity or patronage or pauperism—save the mark!—would be thrust aside and our national honour would be kept bright and pure. We welcome the action of the War Office in announcing through the Censor's Office that each recruit when he joins the Colours is to bring with him his marriage certificate and the birth certificate of each of his children. We may add, for the comfort of our Charity Organisation Society friends and others, that the war already proves that every Britisher who has fought in France lately, like everyone who has joined the Colours, is entitled to be treated as a gentleman and man of honour, and any indiscretion or extravagance, if such there be, amongst his dependants during his absence, until they are helped to realise and work under the new conditions, are trifles representing in the whole a sum of money which is infinitesimal compared with the waste inseparable from war conditions in other directions. The whole nation and Empire, thank God, are one people. The men who have joined, or are joining, the Colours have proved themselves to be the very best type of Britisher which our nation can recruit for the war. Let every other man amongst us forthwith set about doing what we have shown he can do for his country.

### The Politicians Block the Way.

At the Guildhall meeting the politicians, and especially Mr. Bonar Law, pledged themselves to see that the dependants of our sailors and soldiers should be treated as the children of the State. Why are they not being so treated? Is it because the Postmaster-General and his staff block the way by refusing to become the distributing centre in the way we have suggested? The nation wants a Henry Fawcett as Postmaster-General in this emergency, who would prove himself a man of action and uphold the honour of the country towards the dependants of our sailors and soldiers. It rests with the House of Commons before it dissolves to see that arrangements are forthwith made to secure that every payment to every dependant of our warriors is made by the War Office through the Post Office. Then the Lord Mayors, the Mayors, the chairmen of County and District Councils, and representatives of the people throughout Great Britain will, we are certain, gladly do everything else which is needful on the simple plan we have suggested. Remembering Mr. Asquith's speech at the Guildhall, and that Mr. Bonar Law made such a point of the protection of the rights of the homes and the comforts of the dependants of our sailors and soldiers, can they suffer Parliament to dissolve before the Post Office is set in motion and it is so made impossible that there should be any further failure to uphold the independence of the wives and children of our representatives on the seas and on the battlefield?

